# High Incidence of Neonatal Danger Signs and Its Implications for Postnatal Care in Ghana: A Cross-Sectional Study

**DOI:** 10.1371/journal.pone.0130712

**Published:** 2015-06-19

**Authors:** Sumiyo Okawa, Evelyn Korkor Ansah, Keiko Nanishi, Yeetey Enuameh, Akira Shibanuma, Kimiyo Kikuchi, Junko Yasuoka, Margaret Gyapong, Seth Owusu-Agyei, Abraham Rexford Oduro, Gloria Quansah Asare, Abraham Hodgson, Masamine Jimba, Ghana EMBRACE Implementation Research Project Team

**Affiliations:** 1 Department of Community and Global Health, Graduate School of Medicine, The University of Tokyo, Tokyo, Japan; 2 Research and Development Division, Ghana Health Service, Accra, Ghana; 3 Kintampo Health Research Centre, Kintampo, Brong-Ahafo, Ghana; 4 Dodowa Health Research Centre, Dodowa, Greater Accra, Ghana; 5 Navrongo Health Research Centre, Navrongo, Upper East, Ghana; 6 Ghana Health Service, Accra, Ghana; NIH, UNITED STATES

## Abstract

**Background:**

Reducing neonatal mortality is a major public health priority in sub-Saharan Africa. Numerous studies have examined the determinants of neonatal mortality, but few have explored neonatal danger signs which potentially cause morbidity. This study assessed danger signs observed in neonates at birth, determined the correlations of multiple danger signs and complications between neonates and their mothers, and identified factors associated with neonatal danger signs.

**Methods:**

A cross-sectional study was conducted in three sites across Ghana between July and September in 2013. Using two-stage random sampling, we recruited 1,500 pairs of neonates and their mothers who had given birth within the preceding two years. We collected data on their socio-demographic characteristics, utilization of maternal and neonatal health services, and experiences with neonatal danger signs and maternal complications. We calculated the correlations of multiple danger signs and complications between neonates and their mothers, and performed multiple logistic regression analysis to identify factors associated with neonatal danger signs.

**Results:**

More than 25% of the neonates were born with danger signs. At-birth danger signs in neonates were correlated with maternal delivery complications (r = 0.20, p < 0.001), and neonatal complications within the first six weeks of life (r = 0.19, p < 0.001). However, only 29.1% of neonates with danger signs received postnatal care in the first two days, and 52.4% at two weeks of life. In addition to maternal complications during delivery, maternal age less than 20 years, maternal education level lower than secondary school, and fewer than four antenatal care visits significantly predicted neonatal danger signs.

**Conclusions:**

Over a quarter of neonates are born with danger signs. Maternal factors can be used to predict neonatal health condition at birth. Management of maternal health and close medical attention to high-risk neonates are crucial to reduce neonatal morbidity in Ghana.

## Introduction

Sub-Saharan African countries have made significant efforts towards achieving the Millennium Development Goals (MDGs) by 2015. However, many are unlikely to meet the set target [1]. The global under-five mortality rate has declined by half from 1990 to 2013 [1]. The remaining challenge is due to the significant contribution of neonatal deaths to the under-five mortality rate [1]. Thus, saving the lives of neonates should be a key priority in improving child survival and health.

Previous studies have shown that neonatal death is affected by various factors. Maternal factors associated with neonatal death include young maternal age, primi-or grand-multi parity, short birth intervals, maternal health complications, and not breastfeeding [2–10]. Neonatal factors associated with their death were preterm birth, low birth weight, multiple births, and male gender [2, 3, 5–7, 11–13]. The lack of appropriate care during pregnancy, delivery, and the postpartum period respectively [6–9], and residence in rural or poor socio-economic community were also associated with neonatal death [2.6].

While many studies have examined factors contributing to neonatal mortality in resource-limited settings, few studies have focused on neonatal danger signs and complications. These abnormal health conditions could eventually lead to life-threatening complications or death [14]. Mortality risk may be even higher if the neonate and the mother have multiple danger signs or complications. Thus, it is crucial to explore the incidence of neonatal danger signs and complications. When timely care and treatment are provided for the complications, neonatal death might be averted.

Ghana is one of the sub-Saharan African countries with a high neonatal mortality rate. The national MDG target for infant mortality was set at 26 per 1,000 live births [15]. However, it remained at 59 per 1,000 live births in 2010 [15]. In some population surveys conducted in Ghana, the major causes of neonatal death were infections, birth asphyxia and injury, prematurity, and perinatal-related disorders [3, 5, 16, 17]. Since 2000, Ghana has been implementing a three-tiered primary health care system in which the Community-based Health Planning and Service (CHPS) delivers health care and education at the community level, which is the lowest level of health care provision in the country [18]. Under the CHPS strategy, community health officers provide primary health care in homes and at CHPS compounds in collaboration with community volunteers [18]. An initial study in the Northern part of the country indicated that this strategy of service delivery led to a reduction in child mortality [19], potentially through improved quality and increased utilization of health care services at the primary level [20]. A study using the data of the Ghana Demographic and Health Survey (DHS) identified various determinants of neonatal death [6]. However, the incidence of neonatal danger signs during the birth period and their associated factors in Ghana have not been examined.

To address these gaps, this study examined the incidence of danger signs and complications, determined the correlations between multiple danger signs and complications in neonates and their mothers from pregnancy to six weeks postpartum, and identified factors associated with neonatal danger signs at birth.

## Methods

### Study setting

This cross sectional study was conducted as part of a situational analysis of the Ghana Ensure Mothers and Babies Regular Access to Care (EMBRACE) Implementation Research Project [21], a collaboration between the Ghana Health Service (GHS), the University of Tokyo, and the Japan International Cooperation Agency (JICA). GHS oversees three Health Research Centres (HRC) located in the three different eco-epidemiological zones of the country: Navrongo HRC in the Upper East region; Kintampo HRC in the Brong-Ahafo region; and Dodowa HRC in the Greater Accra region. Each of the HRCs runs a Health Demographic Surveillance System (HDSS) covering a total of six districts, which were examined in this study. The HDSS collects data from whole communities over time, monitors new health threats, tracks population changes, and assesses policy interventions.

### Participants and data collection

This study recruited 1,500 pairs of women and their neonates through the HDSS databases of the three HRCs. Two-stage random sampling was subsequently conducted to select 500 eligible pairs of women and their neonates from each HRC site. The target women were all aged between 15 and 49 years, had a resident membership at the study sites on the date of the survey, and got pregnant and delivered a live or stillborn baby between January 2011 and April 2013. If a woman got pregnant and delivered twice or more between January 2011 and April 2013, information pertaining to the most recent pregnancy was used. If a woman had a multiple birth, one child was randomly selected for the interviews. Of the 16 women who had delivered twins, six provided data for both neonates; thus, we randomly selected and excluded data from one of the neonates, as well as the duplicated maternal data (n = 6). Data from the mother-neonate pairs were also excluded from the dataset due to missing key data (n = 3) and miscarriage (n = 1). Thus, data from a total of 1,490 pairs of women and their neonates were used for the analysis, including 13 stillbirths and 15 neonatal deaths within six weeks postpartum.

During the data collection period, trained interviewers visited the homes of the selected women and conducted face-to-face interviews. Using structured questionnaires, the women were asked about their socio-demographic characteristics; utilization of antenatal care (ANC), delivery care, postnatal care (PNC), and medical care; complications that they experienced from the latest pregnancy up until six weeks postpartum; and the danger signs and complications that their neonates showed at birth and within the first six weeks. To ensure validity of the data on the utilization of health and medical care, and history of complications and danger signs, the interviewers asked these questions without prompt, followed by with-prompt, and they were cross-checked with maternal health record book. In addition, the HDSS database was used to acquire information on household assets.

### Independent variables

Potential determinants of neonatal danger signs were categorized into four domains: 1) maternal factors, 2) family factors, 3) antenatal factors, and 4) delivery factors based on the conceptual frameworks of Kayode [6] and Mosley [22]. Maternal factors included age, educational level, marital status, and parity. Family factors included wealth quintile rank, ownership of a valid national health insurance card, family support, and means of transportation to access an ANC clinic. Wealth quintiles were established via principal component analysis based on the ownership of the following household asset items: electricity, source of cooking fuel, toilet facility, sewing machine, radio, television, cooking device, fridge, motorbike, car, and mobile phone. Family support was assessed based on four items that would likely affect a woman’s decision to take her sick neonate to a health facility: financial resources to pay for care, a caretaker for other children, a companion to accompany the woman and her neonate to a health facility, and encouragement from family members to visit a health facility.

Antenatal factors included the total number of antenatal clinic visits and the reception of the following essential antenatal care services: education on complications, nutrition and family planning, tetanus toxoid immunization, intermittent preventive treatment for malaria, and HIV testing. Delivery factors included health complications experienced during delivery and place of delivery.

### Dependent variable

Neonatal danger signs at birth were measured through maternal recall and review of the maternal health record books. The assessed danger signs included cold body, very small size, inability to suck, not crying, fever, difficulty in breathing, preterm birth, and bleeding [23].

### Statistical analysis

Descriptive analyses were performed to examine background characteristics of the women and their neonates, incidence of maternal complications, incidence of danger signs and complications among neonates, and utilization of PNC and health facility visit for treatment. Pearson’s correlation coefficients were calculated to assess the correlations between number of danger signs and complications that women and their neonates had during different periods (i.e. pregnancy, delivery, and postnatal periods). A multiple logistic regression analysis was performed to identify factors associated with neonatal danger signs at birth. In this model, the cluster robust estimate of variance was used to allow within-cluster correlations at the sub-district level.

### Ethical considerations

Ethical approval was obtained from the Research Ethics Committee of the Graduate School of Medicine, the University of Tokyo, the Ethics Review Committee of Ghana Health Service, the Institutional Review Board of Navrongo Health Research Centre, the Institutional Ethics Committee of Kintampo Health Research Centre, and the Institutional Review Board of Dodowa Health Research Centre. These ethical oversight bodies approved the following procedure of informed consent.

Written informed consent was obtained from all participants before the start of the interviews. If an eligible participant was between 15 and 17 years of age, written informed consent was obtained from their parents for study participation in advance. Participant confidentiality was strictly enforced.

## Results

### Basic characteristics of study participants

Table 1 is a summary of the basic characteristics of the participants (n = 1,490). Of all, 39.1% had no education, 61.0% were married, 46.6% had a valid national health insurance membership card, 3.6% received all four types of family support measured, and 49.8% accessed the health facility on foot. Regarding ANC and delivery care factors, 86.1% received ANC four or more than four times, 55.4% received all six essential antenatal services, and 75.6% gave birth at a health facility.

**Table 1:** Summary of participants’ characteristics (n = 1,490).

	n	(%)
***Maternal Factors***		
**Age** (n = 1,473)		
Median (IQR)^✝^	27	(23–33)
≤19	130	(8.8)
20–29	744	(50.5)
30–39	502	(34.1)
40–49	97	(6.6)
**Education**		
None	582	(39.1)
Primary	343	(23.0)
Middle	420	(28.2)
Secondary	111	(7.5)
Tertiary or above	34	(2.3)
**Marital status**		
Married	909	(61.0)
Cohabitating	392	(26.3)
Other	189	(12.7)
**Parity**		
1	393	(26.4)
2–3	631	(42.4)
4–5	289	(19.4)
≥6	177	(11.9)
***Family factors***		
**Wealthy quintile**		
1 (Highest)	298	(20.0)
2	295	(19.8)
3	293	(19.7)
4	305	(20.5)
5 (Lowest)	299	(20.1)
**Valid national health insurance card**		
Own	694	(46.6)
Not own	796	(53.4)
**Family support**		
Yes	54	(3.6)
No	1,436	(96.4)
**Means of access health facility**		
On foot	742	(49.8)
Other	748	(50.2)
***Antenatal factors***		
**ANC attendance**		
≤3	207	(13.9)
≥4	1,283	(86.1)
**ANC essential services**		
≤5 services	665	(44.6)
6 services	825	(55.4)
***Delivery factors***		
**Place of delivery**		
Facility	1,126	(75.6)
Non facility	364	(24.4)

^✝^IQR: Interquartile range

### Complications of mothers and their neonates

As Table 2 shows, 40.5% of the mothers experienced at least one complication during pregnancy, 24.6% at delivery, and 10.5% within six weeks postpartum.

**Table 2:** Maternal Complication (n = 1,490).

	n	(%)
**During pregnancy**		
At least one complication	604	(40.5)
Severe headache	274	(18.4)
Fever	226	(15.2)
Abdominal pain	219	(14.7)
Hyperemesis	215	(14.4)
Swelling feet	137	(9.2)
Severe anaemia	92	(6.2)
Vaginal bleeding	37	(2.5)
Unusual vaginal discharge	35	(2.4)
**During Delivery**		
At least one complication	367	(24.6)
Prolonged delivery	175	(11.7)
Heavy bleeding	113	(7.6)
Premature rupture of the membrane	58	(3.9)
Fever	51	(3.4)
Placenta not expelled 1 hour after birth	34	(2.3)
Compound presentation	33	(2.2)
Multiple-birth	16	(1.1)
Passing urine through vagina	7	(0.5)
Stillbirth	6	(0.4)
Convulsion	4	(0.3)
Rupture uterus	1	(0.1)
**Within 6 weeks postpartum**		
At least one complication	157	(10.5)
Fever	57	(3.8)
Easily felt tired	47	(3.2)
Problem in urination	27	(1.8)
Breast problem	21	(1.4)
Felt breathless during routine household work	16	(1.1)
Severe bleeding	16	(1.1)
Perineum swelling	13	(0.9)
Felt unhappy or crying easily	10	(0.7)
Foul smelling or purulent vaginal discharge	5	(0.3)
Convulsion	4	(0.3)
Passing urine through vagina	1	(0.1)

As Table 3 shows, 26.2% of the neonates exhibited at least one danger sign at birth. Major danger signs were cold body (10.1%), very small size (6.5%), inability to suck (6.1%), not crying (5.5%), fever (4.6%), difficulty breathing (4.6%), preterm birth (1.1%), and bleeding (0.9%). Among liveborn neonates (n = 1,477), 17.7% of the neonates manifested at least one complication within the first six weeks of life.

**Table 3:** Neonatal danger signs and complications.

	n	(%)
**At birth (n = 1,490)**		
At least one danger signs	391	(26.2)
Cold body	151	(10.1)
Very small	97	(6.5)
Too weak to suck/feed	91	(6.1)
Did not cry	82	(5.5)
Had fever	69	(4.6)
Had difficulty in breathing	68	(4.6)
Preterm birth	17	(1.1)
Bleeding	14	(0.9)
**Within 6 weeks of age (n = 1,477)**		
At least one complication	262	(17.7)
Fever	77	(5.2)
Diarrhoea	74	(5.0)
Skin pustules or boils	68	(4.6)
Excess crying	63	(4.3)
Vomiting every feed	46	(3.1)
Not gaining weight	28	(1.9)
Refusal or inability to feed	23	(1.6)
Convulsion	12	(0.8)
Difficulties in breathing	10	(0.7)
Other	43	(2.9)

### Correlations of multiple complications

Table 4 shows correlations between number of danger signs and complications that women and their neonates had from pregnancy to the postpartum period. A positive correlation was found between number of maternal complications during pregnancy and delivery (r = 0.23; p < 0.001). During the delivery period, number of maternal complications was positively correlated with number of danger signs in their neonates (r = 0.20; p < 0.001). A positive correlation was found between number of neonatal danger signs observed during the birth period and complications observed within the first six weeks (r = 0.19; p < 0.001).

**Table 4:** Correlations of multiple complications.

	1	2	3	4	5
1 Maternal complication during pregnancy	1				
2 Maternal complication during delivery	0.23*	1			
3 Maternal complication after delivery	0.23*	0.17*	1		
4 Neonatal danger sign at birth	0.13*	0.20*	0.15*	1	
5 Neonatal complication within 6 weeks of age	0.20*	0.22*	0.22*	0.19*	1

Pearson's correlation coefficient

* p-value < 0.001

### Factors associated with danger signs in neonates

Table 5 shows the factors associated with neonates born with danger signs. Compared with women less than 20 years of age, those aged 20–29 years [adjusted odds ratio (AOR) = 0.62; 95% CI (0.41–0.96)], and 30–39 years [AOR = 0.46; 95% CI (0.30–0.71)] were less likely to have neonates born with danger signs. Compared with women who had tertiary education, women with a lower education level were more likely to have neonates born with danger signs: no education [AOR = 11.96; 95% CI (1.50–95.23)]; primary school [AOR = 11.73; 95% CI (1.61–85.64)]; and middle school [AOR = 9.71; 95% CI (1.29–73.36)]. Women who received ANC less than four times [AOR = 1.78; 95% CI (1.09–2.91)] were more likely to have a neonate born with danger signs than were women who received ANC four or more than four times. Women who had a complication during delivery [AOR = 1.68; 95% CI (1.29–2.20)] were more likely to have a neonate born with a danger sign than those who did not have any complications.

**Table 5:** Factors associated with neonates born with danger signs by multiple logistic regression analysis (n = 1,466).

	Adjusted OR	(95%CI)
***Maternal factors***		
**Age**		
≤19	1	
20–29	0.62	(0.41–0.96)*
30–39	0.46	(0.30–0.71)*
40–49	0.78	(0.37–1.65)
**Education**		
None	11.96	(1.50–95.23)*
Primary	11.73	(1.61–85.64)*
Middle	9.71	(1.29–73.36)*
Secondary	10.35	(0.98–108.97)
Tertiary or above	1	
**Marital status**		
Married	1	
Cohabitating	0.80	(0.54–1.20)
Other	0.78	(0.46–1.31)
**Parity**		
1	1	
2–3	0.73	(0.50–1.07)
4–5	0.82	(0.51–1.31)
≥6	1.11	(0.56–2.20)
***Family factors***		
**Wealthy quartile**		
1 (Highest)	1	
2	0.91	(0.67–1.26)
3	0.81	(0.53–1.24)
4	1.33	(0.86–2.05)
5 (Lowest)	1.49	(1.00–2.22)
**Valid national health insurance card**		
Own	0.83	(0.61–1.14)
Not own	1	
**Family support**		
Yes	1.68	(0.90–3.13)
No	1	
**Means of access health facility**		
On foot	1.03	(0.75–1.42)
Other	1	
***Antenatal factors***		
**ANC attendance**		
≤3	1.78	(1.09–2.91)*
≥4	1	
**ANC essential services**		
≤5 services	1	
6 services	1.00	(0.70–1.43)
***Delivery factor***		
**Maternal complications at delivery**		
Any complications	1.68	(1.29–2.20)*
No complications	1	
**Place of delivery**		
Facility	1.29	(0.91–1.82)
Non facility	1	

* p-value < 0.05

### Utilization of PNC and treatment during the neonatal period

As Table 6 shows, neonates born with danger signs were more likely to visit a health facility within six weeks postpartum (11.9% vs. 20.4%, p < 0.01). However, no significant differences were found between neonates born healthy and neonates born with danger signs in utilization of PNC at two days (24.6% vs. 29.1%, p = 0.08), two weeks (53.0% vs. 52.4%, p = 0.85), or six weeks postpartum (91.5% vs. 92.1%, p = 0.75), respectively. The proportions of PNC utilization were not consistent among the three sites.

**Table 6:** Utilization of PNC and treatment during the neonatal period (n = 1,477).

	Neonates born healthy		Neonates born with danger signs		
	n	(%)	n	(%)	p-value^✝^
**Facility visit for treatment within 6 weeks postpartum**					
All three sites	131	(11.9)	77	(20.4)	<0.01
Dodowa	53	(14.6)	33	(26.6)	<0.01
Kintampo	24	(6.1)	14	(13.7)	0.01
Navrongo	54	(15.7)	30	(19.7)	0.27
**PNC visit at 2 days postpartum**					
All three sites	270	(24.6)	110	(29.1)	0.08
Dodowa	75	(20.7)	33	(26.6)	0.17
Kintampo	70	(17.9)	23	(22.6)	0.28
Navrongo	125	(36.3)	54	(35.5)	0.86
**PNC visit at 2 weeks postpartum**					
All three sites	582	(53.0)	198	(52.4)	0.85
Dodowa	257	(70.8)	83	(66.9)	0.42
Kintampo	180	(45.9)	53	(52.0)	0.28
Navrongo	145	(42.2)	62	(40.8)	0.78
**PNC visit at 6 weeks postpartum**					
All three sites	1,006	(91.5)	348	(92.1)	0.75
Dodowa	333	(91.7)	112	(90.3)	0.63
Kintampo	375	(95.7)	97	(95.1)	0.81
Navrongo	298	(86.6)	139	(91.5)	0.13

^✝^p-value for Chi-square test

## Discussion

We found that more than 25% of neonates were born with one or more danger signs. A unique finding is that, when mothers had complications at delivery, their neonates were also more likely to be born with danger signs. Furthermore, when mothers had multiple complications, their neonates were more likely to show multiple danger signs at birth although the observed correlation coefficients were relatively low. The neonates with multiple danger signs were more likely to develop multiple complications during the neonatal period. Despite the crucial need for postnatal follow-up for neonates with danger signs, PNC uptake in this group was low at two days and two weeks postpartum. Factors associated with danger signs in neonates were teenage pregnancy, educational status less than secondary school, fewer than four ANC visits, and maternal complications during delivery.

These findings indicate that the health status of mothers and their neonates is linked from pregnancy to the postpartum period. Thus, when mothers have any complications during pregnancy, they should be treated before delivery and should give birth at a facility where advanced maternity and obstetric care is available. Additionally, those born with danger signs are in particular need of PNC, as the first 28 days of life is the period of highest risk in a child’s life [24]. However, in this study, a number of neonates with danger signs missed PNC visits during this period, although some might have been hospitalized. Further studies should be conducted to explore the factors contributing to poor uptake of PNC during the neonatal period.

In this study, fewer than four ANC visits resulted in a higher likelihood of neonates being born with danger signs. More than 40% of women had experienced complications during pregnancy, while only 55% of the women had received the six identified components of essential antenatal services. These findings indicate that fewer ANC visits result in delayed diagnosis and management of potential complications. Although the national protocol recommends a minimum of four ANC visits, it is expected that more ANC visits would be scheduled for women who showed signs of complications [23]. It is crucial to ensure that quality antenatal care is provided at the lowest level of health care delivery. The CHPS initiative, a unique primary health system in Ghana, has proven effectiveness in care facilitation, as women who attended CHPS compounds were more likely to receive a series of essential services during pregnancy than were women who lived outside of the CHPS catchment area [20]. Although the government of Ghana is expanding CHPS, huge coverage gaps remain for CHPS health services across the country.

Adolescent women were more likely to give birth to neonates with danger signs than were women aged 20–39 years. Pregnancies in adolescence are known to be associated with increased risk of maternal and neonatal mortality [1, 15]. Ghana ranks high in adolescent fertility [25]. Teenage mothers are more vulnerable owing to psychosocial and financial problems, and lack of parental guidance and support [26–28]. In the post-MDG agenda, adolescent health will be highlighted as an important part of improving life cycle health and well-being [25, 29]. To prevent teenage pregnancy and promote adolescent health, this issue should be addressed in collaboration with non-health sectors [29].

In this study, maternal educational status below the secondary school level was associated with birth of neonates with danger signs. In a previous Ghanaian study, maternal educational status was not associated with neonatal death [6], but a number of studies reported that educational status was a crucial predictor of care-seeking behaviour and maternal literacy for health-related information [30, 31]. In Ghana, the majority of standard maternity-related materials, such as maternal health record books, are written in English. This may limit the ability of some women to understand essential health-related information, resulting in poorer self-care practices and care-seeking behaviour. Thus, pictorial educational materials designed for minimally educated women may facilitate better care-king behaviour, which may reduce adverse health outcomes in neonates.

This study has several limitations. First, the information on complications of women and their neonates relied on their report, which might have been affected by recall bias. To minimize recall bias, we reviewed maternal health record books, and checked for consistency in their responses during the interviews. The validity of the interview data on danger signs and complications might be limited as data were not available which linked them and actual adverse outcomes. However, this study has strength in that we captured the information of service utilizations and complication history from pregnancy to the postpartum period regardless of the health facilities where participants visited; such data cannot be collected in a facility-based survey.

Second, the analysis in this study did not include several important variables, such as birth interval from the previous pregnancy, term of delivery, and neonatal birth weight, as data pertaining to these variables were incomplete or unavailable. However, we did assess several specific variables that were not covered in the national survey. Third, we did not separately analyse the data of the three study sites, which might have limited the identification of site-specific factors. Further analysis considering site-level characteristics will be worth conducting. Finally, this study did not collect data on hospitalization among neonates. Thus, the proportion of PNC uptake in the neonates with danger signs might be underestimated.

## Conclusions

Over a quarter of neonates are born with danger signs. Multiple complications of mothers may increase the risk of giving birth to a child with multiple danger signs and developing further complications during the neonatal period. Neonates do not receive adequate postnatal care in Ghana. Management of maternal health and postnatal follow-up of neonates should be ensured to reduce neonatal morbidity in Ghana.
